# Concept-aware contrastive representation learning for cross-translation semantic consistency modeling of the *Analects*

**DOI:** 10.1371/journal.pone.0358227

**Published:** 2026-09-11

**Authors:** Tang He, Peng Dai, Rong Wang, Yanyang Zhao, Chen Zhao

**Affiliations:** 1 School of Foreign Language Faculty, Chizhou University, Chizhou, China; 2 School of Big Data and Artificial Intelligence, Chizhou University, Chizhou, China; 3 BNRist, Tsinghua University, Beijing, China; 4 School of Cyber Science and Engineering, Ningbo University of Technology, Ningbo, China; Kazan University, RUSSIAN FEDERATION

## Abstract

As a representative Chinese classical text, the *Analects* has been translated into English in many versions over a long historical period, and different translators show substantial variation in concept rendering and language style. To address the lack of a quantifiable and reproducible framework for comparing semantic consistency across multiple English translations of the *Analects*, this paper proposes a concept-aware semantic consistency modeling framework for cross-translation comparison. The proposed method first constructs a unified parallel corpus input based on sentence groups aligned by chapter and sentence IDs. It then introduces concept-term normalization and proper-name normalization to reduce surface lexical variation across translations. In addition, it combines a shared sentence encoder, supervised contrastive learning, and concept-aware constraints to learn sentence representations with cross-translation consistency. Based on the learned representations, the framework is evaluated through two primary tasks, namely cross-translation retrieval and low-consistency detection. Experimental results show that, within the five-translation dataset examined in this study, the proposed method achieves the strongest overall performance across the evaluated settings. The Full model achieves R@1 = 0.936 and F1 = 0.807. The supplementary association analysis further shows that higher model scores tend to be associated with translation pairs labeled as high consistency. These findings support the framework as a computational aid for cross-translation comparison and for identifying translation pairs that may warrant closer expert examination, rather than as a general-purpose method for automatic translation-quality assessment.

## Introduction

In recent years, the large-scale digitization of classical and canonical texts has substantially promoted interdisciplinary integration across linguistics, translation studies, and related fields [1]. This shift has enabled many research questions that traditionally depended on close reading to be reformulated as computable and empirically verifiable problems [2]. As corpora continue to expand and multi-version as well as multi-translation resources become increasingly available, the need for systematic comparison across versions, translations, and even languages is also growing [3]. In this context, natural language processing techniques, particularly deep learning, provide new opportunities for large-scale semantic modeling, cross-text alignment, and generative analysis, thereby opening up new methodological possibilities for digital scholarship on classical texts.

The *Analects*, as a representative Confucian classic, occupies a central position in Chinese intellectual history and educational tradition. Its concise and aphoristic style conveys dense ethical concepts and practical wisdom. This characteristic of “brevity with rich meaning” requires translators, in the process of cross-cultural transfer, to engage in creative interpretation and reconstruction of key concepts and value frameworks. Today, English translations of the *Analects* are numerous and span a wide historical range [4,5]. They also reflect translators’ differing scholarly backgrounds, religious perspectives, and target readership orientations. As a result, the same text has been rendered into multiple diverse and intelligible English versions.

In studies of Chinese classical literature translation, contrastive text analysis is widely used to characterize translator style, evaluate translation quality, and make cross-version differences explicit [6,7]. With the maturation of computer-assisted methods, quantitative evidence has become increasingly important in translation comparison [8,9]. Most prior studies, however, rely on macro-level statistics and surface linguistic features, such as word frequency, sentence length, and rhetorical patterns, to distinguish between translations [10–12]. By contrast, discourse-level studies that explicitly model semantic similarity across translations remain limited, especially for Chinese canonical texts. Semantic similarity measures are well established in NLP [13,14] and provide a natural means of quantifying meaning-level consistency and divergence, thereby enabling scalable and reproducible comparison.

For the *Analects*, existing studies often attribute cross-version differences to translators’ backgrounds and translation strategies [4,5]. However, the supporting evidence is usually drawn from selected examples rather than systematic whole-text benchmarks. Although corpus-based methods improve objectivity, they are rarely combined with task-oriented evaluation, such as retrieval or classification, or with standardized experimental protocols. As a result, method-level comparison and replication remain difficult.

To address these limitations, we introduce semantic representation learning and similarity modeling into the analysis of multiple English translations of the *Analects*. Our goal is to build a quantifiable and reproducible framework for translation comparison. Specifically, we formulate multi-translation comparison as a computable task and propose a semantic consistency modeling framework tailored to the *Analects*. The framework uses sentence-level alignments indexed by chapter and sentence IDs as supervision. It then applies supervised contrastive learning to learn translation-invariant sentence representations, so that different translations of the same saying are pulled closer while different sayings are separated in the semantic space. To improve robustness and interpretability, we further introduce two switchable normalization modules and evaluate their effects through ablation studies. Finally, we instantiate the framework with two primary task-oriented evaluations, enabling controlled and reproducible empirical comparison.

In summary, this paper focuses on multi-translation comparison of the *Analects* and proposes a reproducible framework that integrates digital humanities and natural language processing to quantify semantic consistency and support interpretive analysis. Our main contributions are threefold:

1. We propose a representation learning approach for multi-translation comparison of the *Analects*. The approach uses sentence-level alignments indexed by chapter and sentence IDs as supervision and learns translation-consistent sentence embeddings through supervised contrastive learning. In this way, it robustly aligns parallel translations and quantifies meaning-level divergence.
2. To address variation in key concept terms and variants in person names and proper nouns, we design two controllable normalization modules, namely concept-term normalization and proper-name normalization. We further evaluate their contributions to robustness and interpretability through ablation studies, particularly in low-consistency cases.
3. Based on publicly accessible translations, we construct a reproducible parallel corpus and data processing pipeline for the *Analects*, covering chapter–sentence structure parsing, sentence segmentation, unified indexing, and missing-value placeholders. This resource facilitates future reuse and extension.

The remainder of this paper is organized as follows. Section 2 reviews related work. Section 3 presents the proposed method, including the framework overview, input processing, and concept-aware contrastive learning. Section 4 describes the experimental setup and reports the empirical results. Section 5 discusses the threats to validity. Finally, Section 6 concludes the paper and outlines future work.

## Related work

To support large-scale translation generation and more objective evaluation, machine translation (MT) has gradually developed several major technical paradigms, commonly categorized into four approaches: rule-based MT (RBMT), example-based MT (EBMT), statistical MT (SMT), and neural MT (NMT).

Rule-based machine translation (RBMT) is built on explicit linguistic knowledge, including bilingual lexicons, morpho-syntactic analysis rules, and transfer rules. Although RBMT offers strong controllability and interpretability, it is often limited by the high cost of rule engineering, difficulty in ambiguity resolution, and poor adaptability to domain shifts. Recent studies have therefore explored learning-enhanced and hybrid RBMT frameworks to improve robustness and portability. For example, Islam et al. show that refined linguistic modeling can enable RBMT to outperform some data-driven systems in specific settings [15], while Huang et al. demonstrate that rule–neural hybrid designs can better balance controllability and translation quality [16]. Comparative analyses across RBMT, SMT, and NMT further suggest that RBMT retains value in error-sensitive evaluation, post-editing, and rapid prototyping scenarios [17–20].

Example-based machine translation (EBMT) relies on analogical reasoning by retrieving source segments similar to the input from a bilingual example base or translation memory, and generating translations through fragment substitution and recombination [21,22]. Early studies established the basic EBMT paradigm in terms of retrieval, matching, alignment, and recombination [21,22]. Subsequent research has mainly focused on improving structural representation, meaning preservation, and matching quality. For example, hierarchical representations and structural-semantic constraints have been introduced to improve compositionality and reduce semantic drift during recombination [23–25]. Other studies have enhanced matching and adaptation through fuzzy logic, bilingual lexicons, and ontology-based constraints, particularly in domain-transfer and low-resource settings [26–31].

Statistical machine translation (SMT) is a probabilistic framework that learns translation correspondences from parallel data and performs decoding by combining translation, language, and reordering models. It evolved from early word-alignment methods to phrase-based modeling and log-linear optimization, and many of its core ideas have influenced later neural approaches. Prior work has extended SMT to a variety of settings, including information retrieval [32], unsupervised translation from monolingual corpora [33], and text simplification [34]. Research has also examined SMT optimization in depth, showing that parameter tuning and minimum-risk training are crucial to both performance and stability [35]. At the interface between SMT and NMT, SMT-derived features such as lexicons, phrase tables, and alignment information have been incorporated into neural models to improve translation quality, especially in sparse or alignment-sensitive settings [36], while SMT has also been used as an analytical lens for interpreting NMT training behavior [37].

Neural machine translation (NMT) is an end-to-end paradigm that typically adopts an encoder–decoder architecture, with attention mechanisms and Transformers as the dominant instantiations, to model the conditional generation process from a source sentence to a target sentence. Benefiting from large-scale data and computation, NMT has advanced rapidly, and prior surveys have reviewed its models, resources, and tools while also identifying its practical challenges and deployment risks [1,38–41]. Existing studies have explored several major directions, including scalability, adaptation, controllability, and representation enhancement. For example, large-scale training and scaling-law analyses show that increasing data, model capacity, and compute can substantially improve translation quality, although stable optimization and evaluation remain essential [42,43].

Other work has focused on domain adaptation and controllability, such as scalable adaptation mechanisms, terminology-constrained decoding, and the incorporation of pretrained language models to strengthen translation representations [44–46]. In multilingual and zero-shot settings, massively multilingual models show that parameter sharing can yield substantial transfer gains and enable zero-shot translation [47–49]. Contrastive objectives and multilingual denoising pre-training have also been shown to improve cross-lingual sharing, generalization, and low-resource transfer [50,51], while unsupervised NMT demonstrates that translation mappings can be induced from monolingual corpora without parallel supervision [52].

At the same time, increasing attention has been paid to the reliability and interpretability of NMT, including hallucination analysis, unsupervised quality estimation, and attribution-based interpretation of model predictions [53–56]. Beyond conventional text-to-text translation, NMT has also been extended to multimodal and software-engineering tasks [57–59]. Overall, NMT has become the dominant paradigm in machine translation, with continuing progress in multilingual transfer, low-resource adaptation, controllability, and representation learning. However, existing NMT research is primarily oriented toward translation generation and system-level quality improvement. It pays much less attention to the problem addressed in this study, namely semantic consistency modeling across multiple existing translations of the same classical text. In particular, current methods rarely combine sentence-level alignment, concept-level semantic constraints, and task-oriented evaluation for fine-grained cross-translation comparison. This limitation motivates the framework proposed in the next section.

## Method

### Framework overview

**Fig 1:**
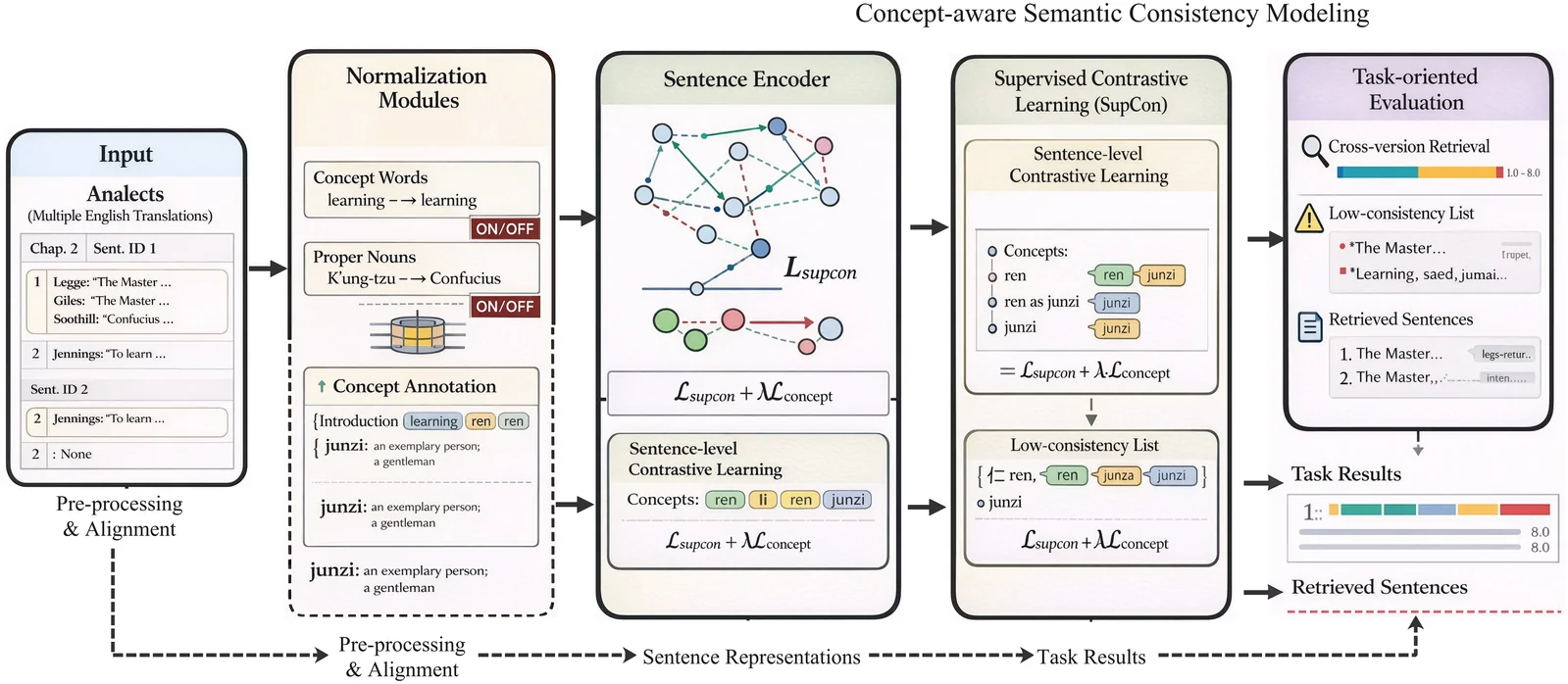
Concept-aware semantic consistency modeling framework for multiple English translations of the *Analects.*

This paper proposes a concept-aware semantic consistency modeling framework for multi-translation comparison of the *Analects*, as illustrated in Fig 1. Given multiple English translations, we first construct sentence-level aligned groups based on shared chapter and sentence IDs. We then perform unified cleaning and structural preprocessing on the input texts. Next, we introduce two optional normalization modules, namely concept-term normalization and proper-noun normalization. These modules reduce surface variation caused by lexical choice and naming differences. The processed sentences are then fed into a shared sentence encoder that maps them into a common semantic representation space. This space serves as the basis for subsequent consistency modeling and discrepancy analysis.

At the representation learning stage, the framework uses sentence-level alignments indexed by the same chapter and sentence IDs as supervision. It applies supervised contrastive learning to bring different translations of the same saying closer in the representation space. It also pushes apart semantically unrelated sayings. In addition, we introduce a concept-aware constraint. This constraint encourages expressions that share the same core concept to remain close across translations. The combination of sentence-level supervision and concept-level constraints enables the framework to capture both local semantic consistency and broader patterns of concept transfer. Finally, we evaluate the learned sentence representations through two primary downstream tasks, together with a supplementary score-label association analysis. This design yields a quantifiable, comparable, and reproducible framework for empirical analysis.

### Input processing and normalization

The input to our framework consists of *M* English translations of the *Analects*, denoted by the translation set $𝒯=\{T^{\left(1\right)},...,T^{\left(M\right)}\}$. To ensure comparability across translations, we use chapter and sentence IDs as a unified index. We then organize the translations into sentence-level aligned groups. Specifically, for each chapter–sentence index (*c*,*i*), we construct a translation group ${𝐱}_{c,i}=\{x_{c,i}^{\left(m\right)}{\}}_{m=1}^{M}$, where $x_{c,i}^{\left(m\right)}$ denotes the sentence from version *T*^(*m*)^ at that position. Because the available translations differ in formatting and completeness, some indices may be missing in certain versions. In such cases, the corresponding position is retained and marked as NULL. Table 1 shows an example of this processing procedure, including the aligned sentence groups, concept tags, and normalized inputs.

**Table 1:** An example of input processing for aligned sentence groups of the *Analects.*

Version	Original Sentence	Concept Tags	Concept-normalized Form
Legge	Is it not pleasant to learn with a constant perseverance and application?	{learning}	learn → learning
Lyall	To learn and then do, is not that a pleasure?	{learning}	learn → learning
Giles	To learn, and to practise on occasion what one has learnt—is this not true pleasure?	{learning}	learn / learnt → learning
Jennings	To learn, said the Master, and then to practise opportunely what one has learnt—does not this bring with it a sense of satisfaction?	{learning}	learn / learnt → learning
Soothill	Is it not indeed a pleasure to acquire knowledge and constantly to exercise oneself therein?	{learning}	acquire knowledge → learning
Legge	Is it not delightful to have friends coming from distant quarters?	{friendship, afar}	friends / distant quarters → friendship, afar
Lyall	When friends come from afar do we not rejoice?	{friendship, afar}	friends / afar → friendship, afar
Giles	The coming of a friend from a far-off land—is this not true joy?	{friendship, afar}	friend / far-off land → friendship, afar
Jennings	To have associates (in study) coming to one from distant parts—does not this also mean pleasure in store?	{friendship, afar}	associates / distant parts → friendship, afar
Soothill	And is it not delightful to have men of kindred spirit come to one from afar?	{friendship, afar}	men of kindred spirit / afar → friendship, afar

At the structural processing stage, we first perform chapter parsing and sentence segmentation for each translation. We then extract the chapter–sentence hierarchy and map each translated segment to the unified index (*c*,*i*). The alignment step follows a hard alignment strategy based on identical chapter and sentence IDs. Hard alignment was applied only when chapter and sentence identifiers were unambiguous. After text extraction and structural recovery, we checked identifier continuity, duplicate records, missing entries, and abnormal text lengths. Particular attention was given to the Giles translation because of its partial and thematic organization. Uncertain or unmatched passages were marked as missing and excluded from positive-pair construction and retrieval evaluation. Based on the aligned groups, sentence-level positive samples are constructed from non-empty translations under the same index, whereas samples from different indices are treated as negative candidates.

To reduce the effect of non-content noise, we further apply unified cleaning and quality control to the aligned sentence groups. First, we remove non-body content and normalize whitespace, punctuation forms, and abnormal characters. Second, we filter anomalous fragments according to length and structural features, such as overly short incomplete sentences, excessively long concatenated sentences, and entries that contain only indices or symbols. Finally, we compute basic corpus statistics, including the number of valid sentence groups, missing rates across translations, and sentence-length distributions. These steps improve the consistency and reproducibility of the input data at the whole-text level.

After cleaning, we construct normalized inputs to reduce the effect of surface variation on cross-translation consistency learning. We use two switchable normalization modules. The first is concept-term normalization. This module maps different renderings of the same Confucian concept to a unified concept label based on a predefined concept lexicon. For example, expressions such as *gentleman*, *superior man*, and related variants can be linked to the same underlying concept tag when they refer to *junzi*.

The concept lexicon was developed as a manually curated expert knowledge resource for the five English translations examined in this study. Candidate lexical variants were collected from the translations and were assigned to the same concept label only when contextual examination indicated that they referred to the same underlying Confucian concept. The resulting lexicon contains 37 concept labels and 266 lexical variants, covering 431 of the 505 aligned groups in the Core Aligned dataset, with a coverage rate of 85.35%. Each concept label contains 7.19 variants on average, and each covered aligned group contains 2.92 concept labels on average. The lexicon was finalized before representation-learning training and was subsequently treated as a fixed deterministic resource. The lexicon contains no consistency labels, split identifiers, similarity scores, or evaluation results, and it was not revised according to validation or test performance. Concept normalization introduces an explicit manually curated expert prior rather than learning concept equivalence through representation optimization. Because lexical variants from all five translations were consulted during lexicon construction, including expressions occurring in held-out groups, this resource should be understood as a closed-corpus expert lexicon rather than a training-only or externally defined lexicon.

The second module is proper-noun normalization. This module handles transliterations, aliases, and spelling variants of named entities. It is designed to reduce artificial divergence caused by differences in romanization or naming conventions across translations. Both modules operate only on the target lexical items. They leave the remaining sentence context unchanged and preserve the original forms for subsequent error analysis and case studies.

In addition to normalization, the framework also records concept annotations for each aligned sentence. These annotations serve two purposes. First, they provide explicit semantic tags for downstream concept-aware supervision. Second, they improve the interpretability of low-consistency cases by making concept-level variation directly traceable. In Table 1, the columns “Concept Tags” and “Concept-normalized Form” correspond to the annotation results and normalized inputs, respectively. After these steps, each non-empty translation sentence is tokenized and converted into a model-readable input sequence using a unified maximum length and truncation strategy.

### Sentence representation learning with supervised contrastive learning

**Fig 2:**
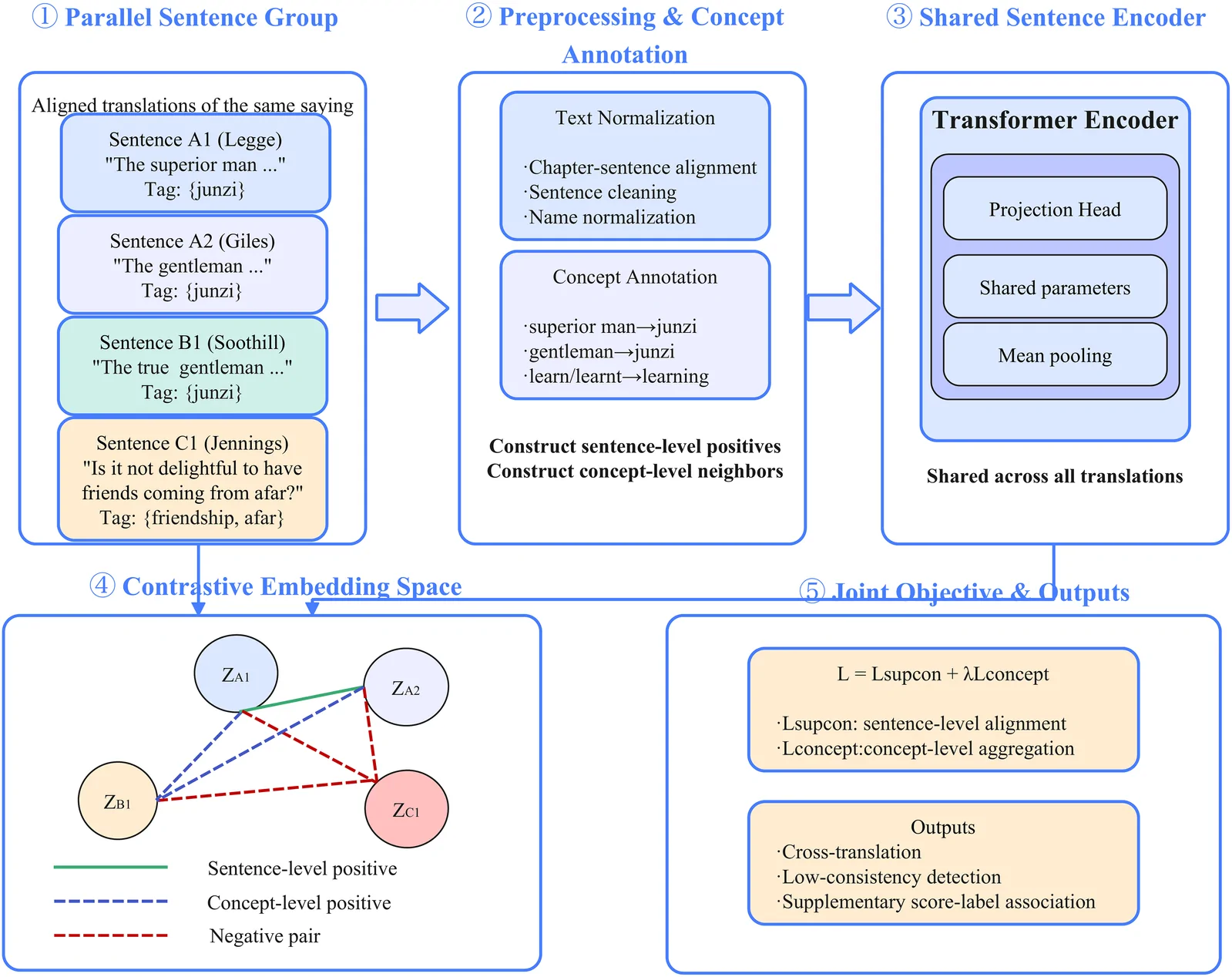
Architecture of the Proposed Concept-aware Contrastive Learning Framework.

After input processing, we use a shared sentence encoder to learn unified representations for aligned translations, as illustrated in Fig 2. Given a non-empty translation sentence $x_{c,i}^{\left(m\right)}$, where *m* indexes the translation version and (*c*,*i*) denotes the chapter and sentence ID, the encoder $f_{\theta }(\cdot )$ first produces a contextual sentence representation $h_{c,i}^{\left(m\right)}\in {\mathbb{R}}^{d}$. This representation is then passed to a lightweight projection head $g_{\phi }(\cdot )$ and normalized to obtain the contrastive representation:


$$\begin{array}{c} h_{c,i}^{\left(m\right)}=f_{\theta }\;\left(x_{c,i}^{\left(m\right)}\right),\;z_{c,i}^{\left(m\right)}=norm\;\left(g_{\phi }\;\left(h_{c,i}^{\left(m\right)}\right)\right). \end{array}$$ (1)

Here, $h_{c,i}^{\left(m\right)}$ denotes the encoder output, and $z_{c,i}^{\left(m\right)}$ denotes the projected representation used for contrastive learning. The encoder parameters are shared across all translation versions. As a result, sentences from different translations are mapped into a common semantic space. This design improves cross-version comparability and provides a unified representation basis for semantic consistency modeling.

The use of a shared encoder is important for multi-translation comparison. Different English translations of the same saying often vary substantially in wording, syntax, and stylistic preference. If each version were modeled independently, the resulting representations would be less directly comparable. By contrast, the shared encoder forces semantically related sentences from different translations to be represented within the same embedding space. The projection head separates the representation used for contrastive optimization from the encoder output. This design facilitates contrastive training while preserving the semantic information encoded by the backbone model. In our setting, it also provides a cleaner representation space for modeling cross-translation alignment.

At the sentence level, we construct a supervised contrastive objective from the aligned sentence groups. For an anchor sample $x_{c,i}^{\left(m\right)}$, all non-empty translations with the same chapter and sentence ID are treated as sentence-level positive samples. These samples correspond to different English renderings of the same source saying. By contrast, samples with different indices $\left(c^{\prime },i^{\prime })\text{≠}(c,i\right)$ are treated as negatives. Let 𝒜 denote the set of valid anchors and *P*(*a*) the sentence-level positive set for anchor *a*. The sentence-level supervised contrastive loss is defined as


$$\begin{array}{c} {ℒ}_{supcon}=\frac{1}{\left|𝒜\right|}\sum_{a\in 𝒜}\left(-\frac{1}{\left|P(a)\right|}\sum_{p\in P(a)}\log\frac{\exp(sim(z_{a},z_{p})/\tau )}{\sum_{b\in ℬ⧵\{a\}}\exp(sim(z_{a},z_{b})/\tau )}\right), \end{array}$$ (2)

where ℬ is the training batch, sim(·,·) denotes cosine similarity, and τ is a temperature parameter. This objective explicitly encourages different translations of the same saying to be close to each other, while pushing apart semantically unrelated sayings. In this way, the model learns translation-consistent sentence embeddings rather than representations dominated by surface lexical variation. This sentence-level contrastive objective forms the basic alignment mechanism of the proposed framework and serves as the foundation for the concept-aware constraint introduced in the next subsection.

### Concept-aware constraint and semantic consistency inference

Although sentence-level supervised contrastive learning can align different translations of the same saying, it does not explicitly model the stability of key concepts across different sentence contexts. This limitation is important for the *Analects*, where many core concepts, such as *ren*, *li*, and *junzi*, recur across sayings and are often rendered in different lexical forms. To further strengthen concept-level semantic consistency, we introduce a concept-aware constraint in addition to sentence-level supervision. Let $𝒞(x_{c,i}^{\left(m\right)})$ denote the set of concept tags associated with sentence $x_{c,i}^{\left(m\right)}$. If two samples share at least one concept tag, they are treated as concept-level semantic neighbors. In this way, the model is encouraged to preserve not only sentence-level alignment for parallel translations, but also concept-level coherence across different sayings.

For an anchor *a*, the concept-level positive set is defined as


$$\begin{array}{c} Q(a)=\{x_{b}\in ℬ|𝒞(x_{b})\cap 𝒞(x_{a})\text{≠}\emptyset ,\;b\text{≠}a\}, \end{array}$$ (3)

where ℬ denotes the current training batch. Based on *Q*(*a*), we define a concept consistency loss ${ℒ}_{concept}$. This loss takes the same contrastive form as ${ℒ}_{supcon}$, but uses concept-sharing neighbors as positives rather than sentences aligned by the same chapter and sentence ID. The final training objective is


$$\begin{array}{c} ℒ={ℒ}_{supcon}+\lambda {ℒ}_{concept}, \end{array}$$ (4)

where λ controls the relative contribution of concept-level supervision. The two losses play complementary roles. The sentence-level loss aligns translations of the same saying. The concept-aware loss promotes coherence among sentences that share core concepts across different contexts. Together, they enable the representation space to capture both local translation alignment and broader patterns of concept transfer.

At inference time, we use the trained shared sentence encoder to generate a final representation for each non-empty translation sentence. Semantic consistency across translations is then measured by cosine similarity in the learned representation space. Specifically, for any two translation sentences $x_{u}$ and $x_{v}$, their consistency score is defined as


$$\begin{array}{c} s(x_{u},x_{v})=sim(r_{u},r_{v})=\frac{r_{u}^{⊤}r_{v}}{\left\|r_{u}\|\|r_{v}\right\|}, \end{array}$$ (5)

where $r_{u}$ and $r_{v}$ denote the $\ell_{2}$-normalized encoder representations used for downstream evaluation. A higher score indicates stronger semantic proximity between two translations. This score provides a unified basis for cross-translation retrieval and low-consistency detection. It is also used in a supplementary analysis of the association between continuous similarity scores and binary human labels.

## Experiments

### Dataset construction

The dataset used in this study consists of five publicly available English translations of the *Analects*, namely those by James Legge (https://www.gutenberg.org/ebooks/3330), Leonard A. Lyall (https://www.gutenberg.org/ebooks/24055), Lionel Giles (https://www.gutenberg.org/ebooks/46389), William Jennings (https://archive.org/details/dli.ministry.25525), and William Edward Soothill (https://archive.org/details/analectsofconfuc00confrich). Table 2 summarizes the source formats and coverage of these translations. All versions are obtained from openly accessible digital sources. They provide representative material for modeling semantic consistency across multiple English translations of the *Analects*. Among them, the versions by Legge, Lyall, Jennings, and Soothill are complete translations of the full text, whereas Giles’s version adopts a selective and thematically reorganized format.

**Table 2:** Source information of the English translations used in this study.

Version	Translator	File Type	Coverage
Legge	James Legge	TXT	Full translation
Lyall	Leonard A. Lyall	TXT	Full translation
Giles	Lionel Giles	TXT	Partial / thematic selection
Jennings	William Jennings	PDF/OCR	Full translation
Soothill	William Edward Soothill	PDF/OCR	Full translation

To construct a computable parallel corpus, we first perform version-level structural parsing on the raw texts. For versions with relatively clear chapter structure, we establish a unified index and map each translation onto a shared chapter–sentence skeleton. For versions with substantial noise or different editorial formats, we first conduct main-text extraction, paragraph recovery, and chapter localization, and then perform sentence segmentation and index alignment. To ensure cross-translation comparability, we adopt a hard alignment strategy based on identical chapter and sentence IDs. If an entry is missing or cannot be reliably aligned, its index position is retained and marked as NULL, thereby preserving a unified whole-text structure. The corpus contains 505 aligned chapter–sentence groups. Legge contains 497 valid groups and 8 missing groups, corresponding to a missing rate of 1.58%. Lyall contains 504 valid groups and 1 missing group, with a missing rate of 0.20%. Confidence filtering retained 311 Giles groups, 468 Jennings groups, and 500 Soothill groups, corresponding to missing rates of 38.42%, 7.33%, and 0.99%, respectively. Across the ten translation-pair combinations, the retained data yield 4,104 pair instances. The aligned groups were divided into training, validation, and test sets using a 70%/15%/15% split. The 70%/15%/15% partition controls the data used for representation learning and model selection. The manually curated concept lexicon was not estimated from these representation-learning subsets. Instead, it was finalized as a fixed expert resource before model training. Test labels, model predictions, and evaluation results were not used in its construction or revision.

Based on the aligned corpus, we further apply unified cleaning and normalization procedures. First, we remove obvious non-body content and standardize whitespace, punctuation forms, and abnormal characters to reduce noise introduced by different digitization sources. Second, we filter anomalous fragments according to length and structural features. Third, we annotate core Confucian concepts using a predefined concept lexicon and normalize variants of person names and proper nouns. This processing pipeline preserves both the original expressions and their normalized forms in the final dataset, thereby supporting reproducible comparison and downstream analysis. The processed dataset and associated materials underlying the findings are publicly available in the Zenodo repository at https://doi.org/10.5281/zenodo.20660361.

### Baselines

To comprehensively evaluate the proposed method, we construct a baseline suite from three perspectives: traditional text similarity methods, embedding-based semantic similarity methods, and internal representation-learning baselines with ablation variants. This design serves two purposes. First, it covers different modeling levels, ranging from surface lexical matching to contextual semantic representation. This allows us to assess the overall advantage of the proposed framework over existing similarity-based methods. Second, through internal comparisons and module ablations, it helps determine whether the performance gains mainly come from sentence-level supervised contrastive learning or from the concept-aware constraint and normalization design.

As shown in Table 3, the external baselines include traditional similarity methods and embedding-based semantic models. TF-IDF and SimHash serve as traditional baselines. The embedding-based baselines include Word2Vec [60], GloVe [61], BERT [62], Sentence-BERT [63], and SimCSE [64]. Word2Vec and GloVe represent static word embeddings, whereas BERT uses contextual representations with mean pooling. Sentence-BERT and SimCSE provide stronger sentence-level representations through contrastive pretraining. Both models are evaluated using publicly available pretrained checkpoints without task-specific fine-tuning. Word2Vec, GloVe, and BERT have also been used in previous studies of semantic comparison among English translations of the *Analects*, providing direct external reference points. For all embedding-based baselines, sentence representations are normalized and compared using cosine similarity under the same data splits and evaluation protocols.

**Table 3:** Baseline methods used in the experiments.

Category	Method	Description
Traditional similarity	TF-IDF	Cosine similarity computed over TF-IDF sentence vectors.
Traditional similarity	SimHash	Hash-based text similarity baseline.
Embedding-based similarity	Word2Vec	Static word embedding baseline with sentence-level similarity.
Embedding-based similarity	GloVe	Static word embedding baseline with sentence-level similarity.
Embedding-based similarity	BERT	Contextual embedding baseline with sentence-level similarity.
Embedding-based similarity	Sentence-BERT	Pretrained all-mpnet-base-v2 sentence embeddings
Embedding-based similarity	SimCSE	Pretrained supervised SimCSE sentence embeddings with cosine similarity.
Representation learning	Encoder-only	Shared sentence encoder without contrastive training.
Representation learning	SupCon	Sentence-level supervised contrastive learning without concept-aware supervision.
Ablation	w/o concept normalization	Full framework without concept-term normalization.
Ablation	w/o name normalization	Full framework without proper-name normalization.
Ablation	w/o concept-aware loss	Full framework without concept consistency loss.
**Proposed method**	Full model	Concept-aware contrastive learning with both normalization modules and the joint objective.

For internal comparisons, we further include representation-learning and ablation baselines. Encoder-only denotes the setting in which only the shared sentence encoder is used to generate sentence representations, and cosine similarity is computed directly without contrastive training. SupCon denotes the setting that uses only sentence-level supervised contrastive learning, without the concept-aware constraint. We also consider three ablation variants, namely without concept normalization (w/o concept normalization), without name normalization (w/o name normalization), and without the concept-aware loss (w/o concept-aware loss). These variants are used to evaluate the contributions of concept-term normalization, proper-name normalization, and the concept consistency loss, respectively. These internal baselines allow us to analyze the role of each component in cross-translation semantic consistency modeling and to verify the necessity of the full model design.

### Experimental settings

The overall experimental setup is illustrated in Table 4. We construct the training, validation, and test sets on a unified chapter–sentence indexing scheme, where all translated sentences associated with the same chapter–sentence index are always assigned to the same data split in order to avoid information leakage. On this basis, we conduct two primary task-oriented evaluations, namely cross-translation retrieval and low-consistency detection, together with a supplementary score-label association analysis. In cross-translation retrieval, a sentence from one translation is used as the query, and the goal is to retrieve the sentence with the same chapter and sentence ID from the candidate set of the other translations. All translated sentences associated with the same chapter–sentence ID were assigned to the same split. This group-level partition prevents sentences corresponding to the same source saying from appearing in different representation-learning subsets. The representation-learning test split was excluded from model training and representation-level hyperparameter selection.

**Table 4:** Experimental settings of the proposed model.

Item	Value
Backbone encoder	bert-base-uncased
Pooling strategy	Mean pooling
Projection head	2-layer MLP
Hidden size of projection head	256
Maximum sequence length	128
Batch size	32
Optimizer	AdamW
Learning rate	$2\times 10^{-5}$
Weight decay	0.01
Temperature τ	0.07
Concept loss weight λ	0.2
Maximum epochs	10
Early stopping patience	3 epochs
Similarity function	Cosine similarity
Runs	5
Framework	PyTorch + Transformers
Hardware	Single NVIDIA GPU

Low-consistency detection was evaluated using 300 human-annotated translation pairs sampled exclusively from chapter–sentence groups in the held-out representation-learning test split. Consequently, neither the annotated pairs nor any sentence from their corresponding aligned groups was included in representation-learning training or validation. Two annotators with backgrounds in translation studies and classical Chinese texts independently labeled each pair as high consistency or low consistency. They were blind to all model outputs and similarity scores. Spearman’s ρ and Kendall’s $\tau_{b}$ were used to measure the association between the continuous model scores and these binary labels. The annotators agreed on 275 pairs (91.67%), with Cohen’s κ=0.833, and disagreements were resolved through adjudication. The 300 annotated pairs were further divided into a human-validation subset and a human-test subset using a 67/233 group-disjoint partition. All pairs derived from the same chapter–sentence group were assigned to the same subset. The human-validation subset was used only to select the decision threshold by maximizing F1. The selected threshold was then fixed and applied to the human-test subset. Accuracy, F1, and AUC were calculated on the human-test subset.

At the implementation level, we adopt a representation learning model composed of a shared sentence encoder and a lightweight projection head. The backbone encoder is bert-base-uncased, and its output is mean-pooled to form sentence-level representations. The projection head is implemented as a two-layer feed-forward network that maps sentence representations into the contrastive learning space. The maximum input length is set to 128 in order to balance the sentence length of classical translations and training efficiency. Model training uses the AdamW optimizer, with an initial learning rate of $2\times 10^{-5}$, a weight decay coefficient of 0.01, and a batch size of 32. Both the supervised contrastive loss and the concept consistency loss use cosine similarity, with the temperature parameter set to 0.07 and the concept loss weight λ set to 0.2. Training is conducted for at most 10 epochs.

To ensure fair comparison among baselines, we use the same data splits and the same sentence-level similarity computation for all embedding-based and representation-learning methods. For internal comparisons and ablation experiments, all settings remain unchanged except for the removed module, including the backbone encoder, input length, optimizer, learning rate, and batch size. For external baselines such as TF-IDF, SimHash, Word2Vec, GloVe, and BERT, we follow either standard implementations or commonly used settings in related work as closely as possible, so as to ensure the interpretability of the comparison results. Sentence-BERT and SimCSE were evaluated using their publicly available pretrained checkpoints without additional fine-tuning.

For evaluation metrics, cross-translation retrieval is evaluated using Recall@1, Recall@5, and MRR, whereas low-consistency detection is evaluated using Accuracy, F1, and AUC. Spearman’s ρ and Kendall’s $\tau_{b}$ are additionally reported as supplementary measures of association between continuous model scores and binary human labels. To reduce the effect of random initialization, all trainable neural models were run five times using the same data split and matched random seeds. We additionally conducted group-level paired permutation tests over the held-out evaluation instances. For retrieval, all queries derived from the same chapter–sentence group were treated as one cluster. For the human-based evaluation, all annotated pairs associated with the same chapter–sentence group in the human-test subset were kept together. For descriptive run-level reporting, we present the mean, standard deviation, and 95% confidence interval across five matched runs. Inferential comparisons between the Full model and SupCon were conducted using 10,000 two-sided group-level paired permutations over the held-out evaluation instances. Holm correction was applied across the three representative metrics. Unless otherwise stated, all experiments are conducted on a single NVIDIA GPU, and the models are implemented with PyTorch and HuggingFace Transformers.

## Main results

**Fig 3:**
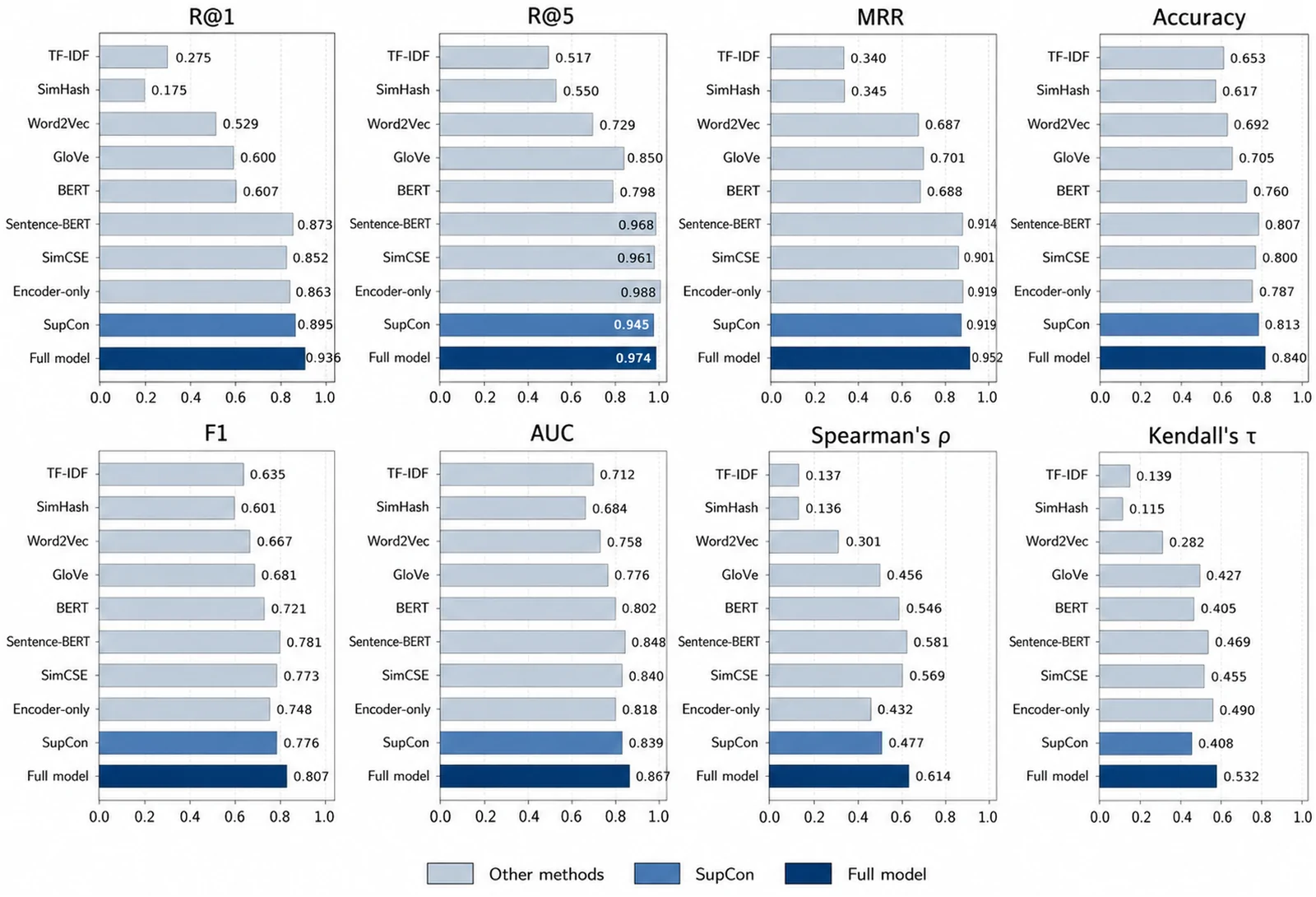
Main results of different methods on the five-translation sentence-level dataset.

We begin with the main results on the five-translation sentence-level dataset. Fig 3 reports the performance of all compared methods on eight evaluation metrics. These metrics cover two primary evaluation tasks and a supplementary association analysis. The comparison provides an overall view of the effectiveness of the proposed method relative to traditional similarity models, embedding-based baselines, and contrastive learning variants.

Overall, Fig 3 shows a clear progression from lexical matching to sentence-level representation learning. Traditional similarity methods perform the worst. TF-IDF achieves only R@1 = 0.275 and MRR = 0.340, while SimHash yields R@1 = 0.175. Static embedding methods provide noticeable improvements. Word2Vec raises R@1 to 0.529, and GloVe further improves it to 0.600. BERT achieves R@1 = 0.607, Accuracy = 0.760, and Spearman’s ρ=0.546. The modern sentence embedding baselines perform substantially better. Sentence-BERT achieves R@1 = 0.873, R@5 = 0.968, and MRR = 0.914, while SimCSE obtains 0.852, 0.961, and 0.901, respectively. Among the representation-learning methods, the Full model achieves the best results on seven of the eight metrics, including R@1 = 0.936, MRR = 0.952, Accuracy = 0.840, F1 = 0.807, AUC = 0.867, Spearman’s ρ=0.614, and Kendall’s $\tau_{b}=0.532$. Encoder-only achieves the highest R@5 of 0.988, whereas the Full model obtains 0.974.

The Full model also maintains clear advantages over the stronger pretrained sentence embedding baselines. Compared with Sentence-BERT, it improves R@1 from 0.873 to 0.936, MRR from 0.914 to 0.952, Accuracy from 0.807 to 0.840, F1 from 0.781 to 0.807, and AUC from 0.848 to 0.867. Spearman’s ρ also increases from 0.581 to 0.614. Similar gains are observed over SimCSE. Compared with SupCon, the Full model improves R@1 from 0.895 to 0.936, Accuracy from 0.813 to 0.840, F1 from 0.776 to 0.807, and AUC from 0.839 to 0.867. These gains indicate that concept normalization and concept-aware supervision provide additional benefits beyond sentence-level contrastive learning alone.

Table 5 summarizes both run-level variability and group-level statistical comparisons. Across the five matched runs, the Full model achieves higher mean R@1, F1, and AUC than SupCon. The additional group-level paired permutation tests yield Holm-adjusted *p*-values of *p* < 0.001, *p* = 0.018, and *p* = 0.044 for R@1, F1, and AUC, respectively. These results provide supplementary evidence that the observed improvements remain evident at the level of held-out chapter–sentence groups and annotated translation pairs, rather than being supported solely by comparisons across five random-seed runs.

**Table 5:** Statistical reliability analysis of representative results. Run-level values are reported as mean ± standard deviation over five matched runs, with 95% confidence intervals for the mean.

Metric	Method	Mean ± Std.	95% CI	Holm-adjusted permutation *p*
R@1	BERT	0.607	–	–
	SupCon	0.895±0.009	[0.884, 0.906]	*p* < 0.001
	Full model	0.936±0.006	[0.928, 0.944]	–
F1	BERT	0.721	–	–
	SupCon	0.776±0.014	[0.758, 0.793]	*p* = 0.018
	Full model	0.807±0.011	[0.793, 0.820]	–
AUC	BERT	0.802	–	–
	SupCon	0.839±0.012	[0.824, 0.854]	*p* = 0.044
	Full model	0.867±0.009	[0.856, 0.878]	–

*Note:* The permutation *p*-values compare SupCon with the Full model using 10,000 two-sided group-level paired permutations over the held-out evaluation instances. All queries or annotated pairs derived from the same chapter–sentence group were treated as one cluster.

To address the potential circularity in low-consistency detection, we further evaluated the compared models on the held-out, separately human-annotated subset. As shown in Table 6, the Full model achieves the best performance, with an Accuracy of 0.840, an F1 score of 0.807, and an AUC of 0.867. These results indicate that the framework can help prioritize translation pairs whose semantic consistency may require closer examination. However, the predicted labels should be treated as computational signals for expert review rather than as automatic or definitive judgments of translation quality.

**Table 6:** Low-consistency detection results on the held-out human-test subset.

Method	Accuracy	F1	AUC
BERT	0.760	0.721	0.802
Encoder-only	0.787	0.748	0.818
SupCon	0.813	0.776	0.839
Full model	0.840	0.807	0.867

**Fig 4:**
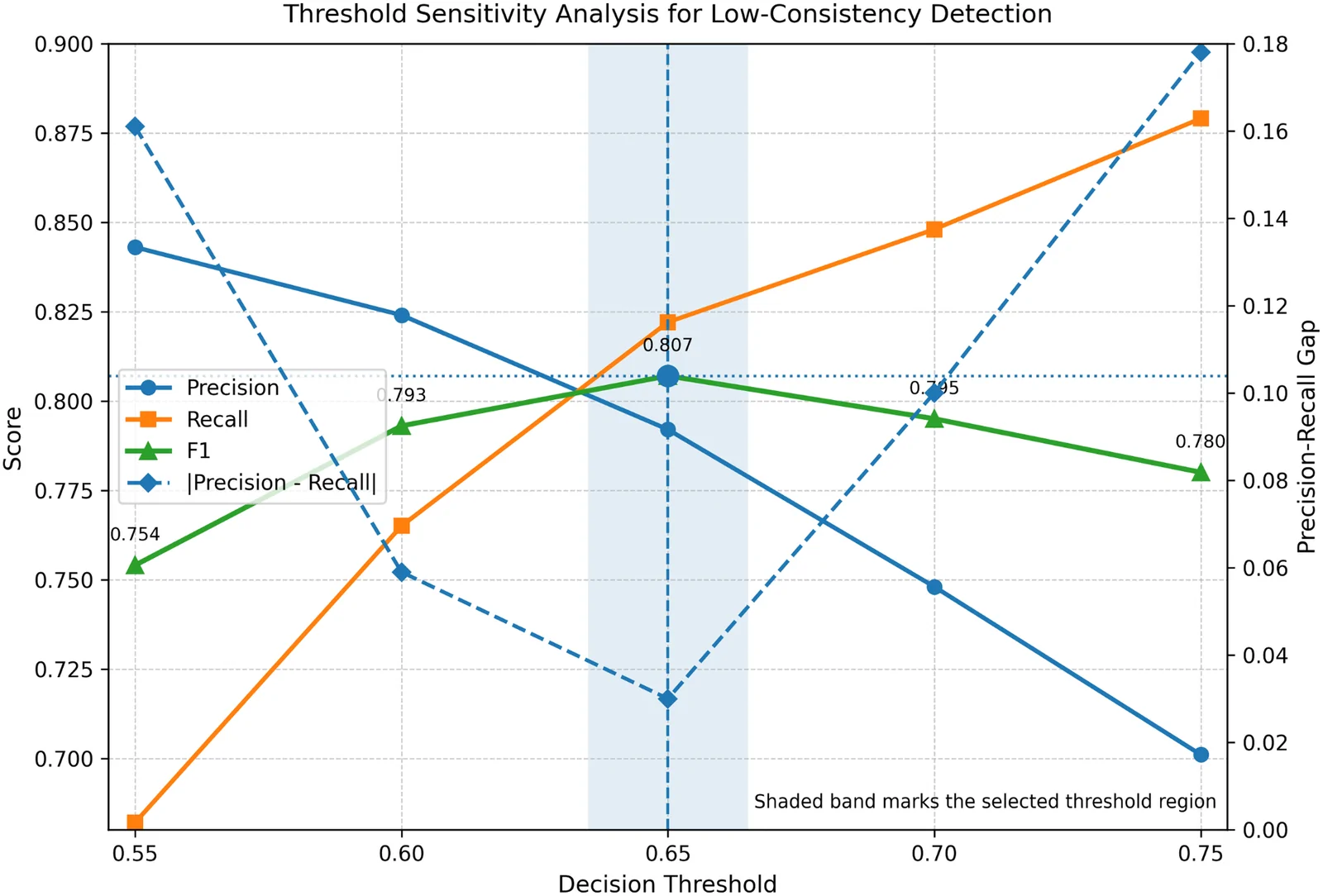
Threshold sensitivity analysis on the human-validation subset.

Fig 4 presents the threshold sensitivity results for low-consistency detection. Precision decreases with increasing thresholds, whereas recall increases. The F1 score peaks at 0.807 when the threshold is set to 0.65. This threshold also produces the smallest precision–recall gap. Moreover, the stable F1 scores between 0.60 and 0.70 indicate that the model is not overly sensitive to threshold selection.

Considering the structural asymmetry of the Giles translation, we further report per-translation retrieval performance in Table 7. The Full model performs consistently well on the four complete translations, with R@1 values ranging from 0.938 to 0.953. The Giles subset obtains a lower R@1 of 0.903, suggesting that its partial and thematic structure introduces additional difficulty. The complete-version average is 0.945, slightly higher than the overall average of 0.936. Thus, Giles does not inflate the overall performance but slightly lowers the average retrieval result.

**Table 7:** Per-translation retrieval performance of the Full model.

Query translation	R@1	R@5	MRR
Legge	0.945	0.982	0.962
Lyall	0.942	0.976	0.955
Giles	0.903	0.941	0.926
Jennings	0.938	0.971	0.949
Soothill	0.953	1.000	0.970
Complete-version average	0.945	0.982	0.959
Overall average	0.936	0.974	0.952

The Full model shows the strongest association with the binary human labels, with Spearman’s ρ = 0.6137 and Kendall’s $\tau_{b}$ = 0.5322 on the human-test subset. These results indicate that higher model scores tend to be assigned to pairs labeled as high consistency. However, the coefficients should not be interpreted as agreement with a graded human ranking. They provide only supplementary evidence of score-based separation between the two consistency classes.

### Ablation study

**Fig 5:**
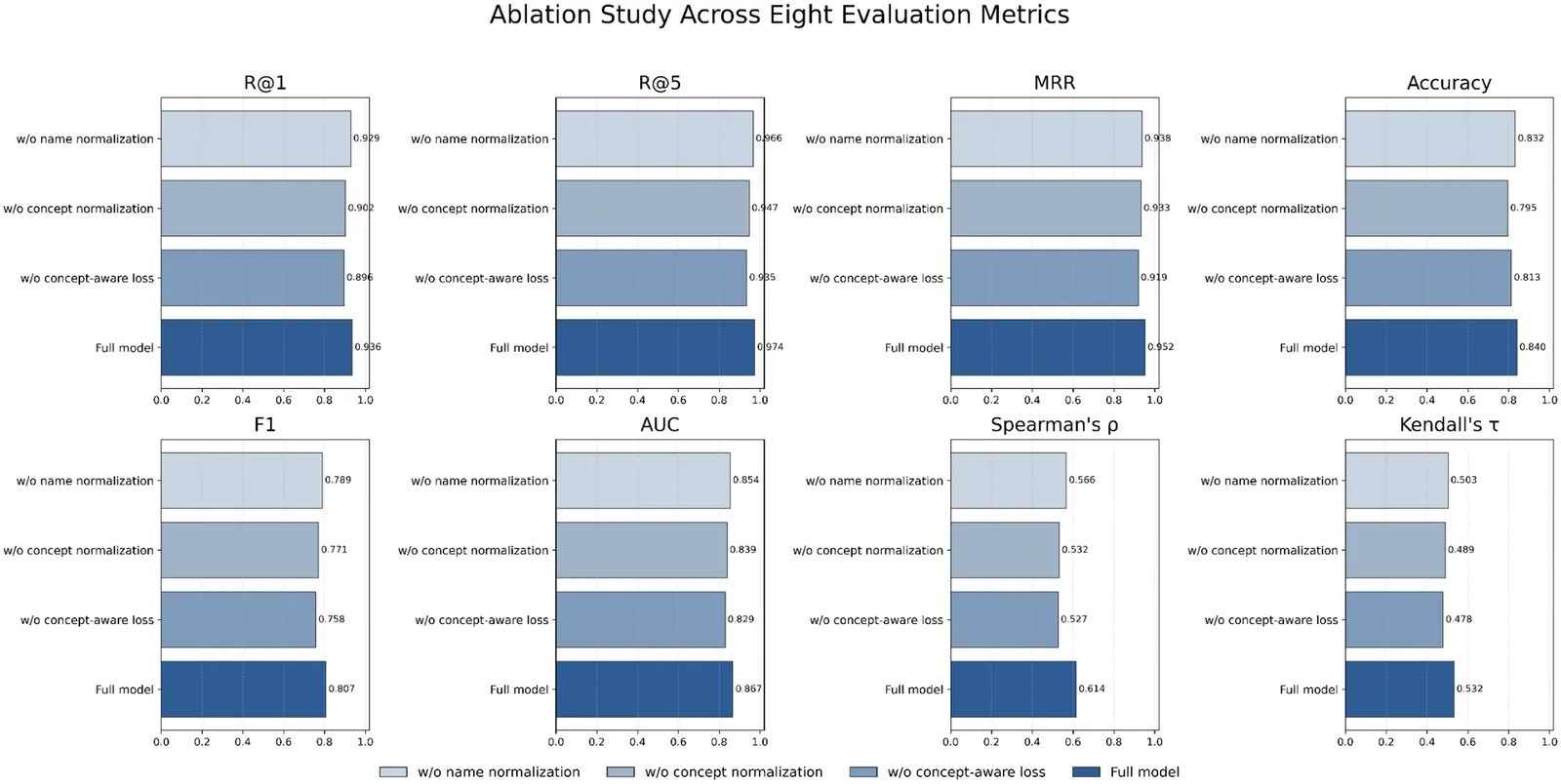
Ablation Study Across Eight Evaluation Metrics on the Translation Dataset.

To analyze the contribution of each component to the final performance, we further conduct an ablation study on the sentence-level dataset, as shown in Fig 5. Overall, the Full model achieves the best results on all eight evaluation metrics. This result indicates that concept normalization, name normalization, and the concept-aware loss all contribute positively to semantic consistency modeling across translations. Specifically, the Full model attains 0.936 on R@1, 0.952 on MRR, 0.807 on F1, 0.867 on AUC, 0.614 on Spearman’s ρ, and 0.532 on Kendall’s $\tau_{b}$.

Comparing the two normalization modules, removing name normalization causes a relatively small performance decrease. For example, R@1 decreases from 0.936 to 0.929, F1 from 0.807 to 0.789, and Spearman’s ρ from 0.614 to 0.566. Removing concept normalization leads to a larger decline. R@1 drops from 0.936 to 0.902, Accuracy from 0.840 to 0.795, F1 from 0.807 to 0.771, AUC from 0.867 to 0.839, and Spearman’s ρ from 0.614 to 0.532. These results confirm the contribution of the explicit concept prior. However, the model retains substantial performance without this module. Thus, the overall gains arise from both representation learning and concept normalization rather than from the encoder alone.

Removing the concept-aware loss causes the largest overall performance decline. Compared with the Full model, this variant reduces R@1 from 0.936 to 0.896, MRR from 0.952 to 0.919, Accuracy from 0.840 to 0.813, F1 from 0.807 to 0.758, AUC from 0.867 to 0.829, Spearman’s ρ from 0.614 to 0.527, and Kendall’s $\tau_{b}$ from 0.532 to 0.478. These declines indicate that concept-aware supervision improves retrieval and low-consistency detection and strengthens the association between model scores and the binary human labels. The ablation without concept normalization further shows that the manually curated lexicon contributes to performance, while the model retains substantial effectiveness without this resource. The reported results therefore reflect contributions from both representation learning and the explicit expert prior, rather than reliance on the lexicon alone.

### Hyperparameter sensitivity analysis

**Fig 6:**
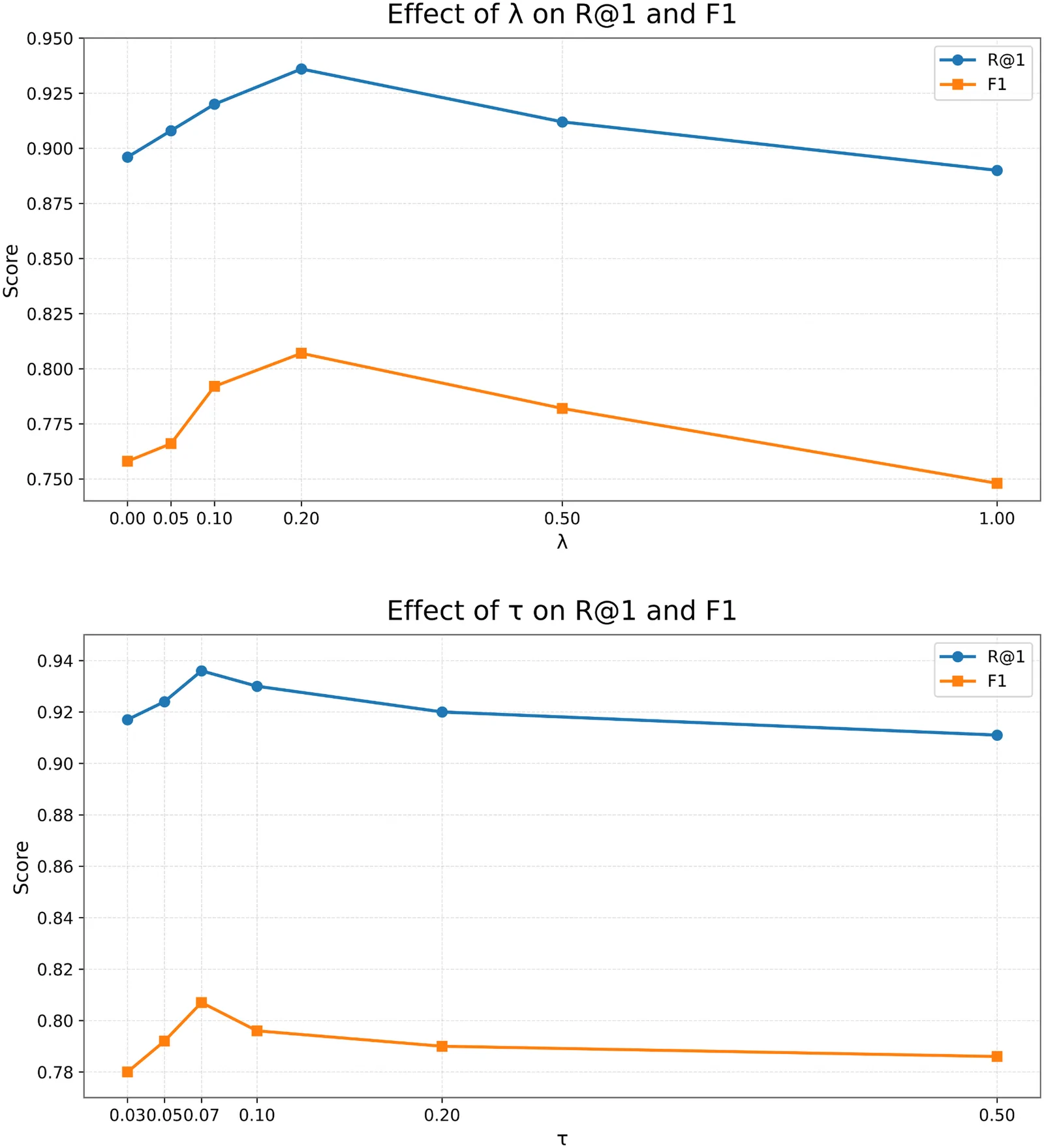
Sensitivity of the Full Model to the Hyperparameters λ and τ.

To evaluate the sensitivity of the proposed method to key hyperparameters, we further examine the effects of the concept-aware loss weight λ and the temperature parameter τ on model performance. We select R@1 and F1 as the main evaluation metrics because they are representative of cross-translation retrieval and low-consistency detection, respectively. This analysis verifies the reasonableness of the default hyperparameter settings. All default representation-level hyperparameters were fixed before evaluation on the human-test subset. The analysis in Fig 6 was conducted post hoc to assess sensitivity and was not used for model selection.

For the concept-aware loss weight λ, performance first improves and then declines as λ increases. When λ=0, the model is equivalent to the *w/o concept-aware loss* variant. As λ increases from 0 to 0.2, R@1 improves from 0.896 to 0.936, while F1 increases from 0.758 to 0.807. This result shows that moderate concept-level supervision benefits both retrieval and low-consistency detection. However, larger values of λ reduce both metrics. An overly strong concept-aware constraint may weaken sentence-level alignment and impair overall performance.

The temperature parameter τ shows a similar pattern. As τ increases from 0.03 to 0.07, R@1 rises from 0.917 to 0.936, and F1 reaches its highest value of 0.807. Further increases in τ gradually reduce both metrics. A large temperature produces a smoother similarity distribution and weakens the distinction between positive and negative samples. The prespecified settings λ=0.2 and τ=0.07 lie near the observed performance peak in this post hoc sensitivity analysis.

### Case study

To further understand how the proposed framework behaves on concrete translation instances, we conduct a qualitative case study on several representative sentence groups from the translation dataset. As shown in Table 8, the selected examples cover three typical situations: semantically consistent renderings with substantial lexical variation, concept-centered translation divergence, and boundary cases involving interpretive paraphrase. These cases complement the aggregate evaluation results and allow us to examine how concept-aware semantic consistency modeling operates at the level of individual sayings.

**Table 8:** Representative case studies for cross-translation semantic consistency analysis.

Case	Concept Tag(s)	Translation Variants	Observation
C1	learning	**Legge**: *learn with a constant perseverance and application*; **Lyall**: *To learn and then do*; **Jennings**: *to practise opportunely what one has learnt*; **Soothill**: *acquire knowledge and constantly exercise oneself therein*.	All versions express the same core idea of *learning*, but differ substantially in wording (*learn*, *do*, *practise*, *acquire knowledge*).
C2	friendship, afar	**Legge**: *friends coming from distant quarters*; **Lyall**: *friends come from afar*; **Jennings**: *associates (in study) coming to one from distant parts*; **Soothill**: *men of kindred spirit come to one from afar*.	This is a high-consistency case with strong lexical divergence. The translations vary from *friends* to *associates* and *men of kindred spirit*, while preserving the shared meaning of like-minded companions arriving from afar.
C3	junzi	**Legge**: *a man of complete virtue*; **Lyall**: *a gentleman*; **Jennings**: *men of the superior order*; **Soothill**: *a true philosopher*.	This case reflects a typical concept-translation divergence centered on *junzi*. While all versions refer to the same source concept, they project it into different semantic frames, ranging from virtue and social status to philosophical identity.
C4	junzi / semantic reinterpretation	The same source concept is rendered with partially overlapping but non-identical semantic emphases, e.g., *gentleman*, *superior man*, *true philosopher*, or *man of complete virtue*.	This type of case remains challenging even for the full model. Although concept-aware supervision improves alignment, highly interpretive translations may still blur the boundary between faithful conceptual transfer and semantic reinterpretation.

Cases C1 and C2 illustrate situations in which the translations remain highly consistent in meaning despite substantial surface variation. In C1, the shared concept of *learning* is expressed through different lexical realizations, such as *learn*, *do*, *practise*, and *acquire knowledge*. In C2, the idea of companions arriving from afar is rendered as *friends*, *associates*, or *men of kindred spirit*. These examples show that lexical overlap alone is not sufficient for reliable comparison. Semantically equivalent translations may differ greatly in wording. The proposed framework handles such cases more effectively because concept normalization reduces lexical mismatch, while concept-aware supervision keeps semantically aligned renderings close in the representation space.

Case C3 and the boundary case C4 further show why concept-level modeling is necessary, while also revealing its current limitations. In C3, the source concept *junzi* is translated as *a man of complete virtue*, *a gentleman*, *men of the superior order*, and *a true philosopher*. These renderings reflect different interpretive choices rather than simple lexical variation. This case highlights the importance of explicit concept-aware learning, because surface-form similarity alone cannot reliably capture the semantic continuity among such expressions. At the same time, C4 shows that highly interpretive translations remain challenging even for the Full model. When a translation shifts from conceptual transfer to broader explanatory paraphrase, the boundary between semantic equivalence and semantic reinterpretation becomes less clear. This observation suggests that, although concept-aware supervision substantially improves cross-translation comparison, modeling deeper interpretive divergence in classical texts remains an important direction for future work.

### Threats to validity

**Internal validity.** The internal validity of this study is mainly influenced by the data processing pipeline and the experimental setup. First, all English translations are obtained from publicly accessible digital resources. The reconstruction of the Jennings and Soothill texts involves OCR cleaning, main-text extraction, and structural recovery. These steps may introduce a limited amount of noise or alignment error. Second, sentence-level alignment mainly relies on chapter/sentence IDs and rule-based recovery. Although missing entries and anomalous fragments are handled in a unified manner, the effect of local mismatches cannot be fully excluded. In addition, the reported results may still be affected by parameter settings, random initialization, and implementation differences. The ablation study measures the overall contribution of the concept prior, but it does not establish whether the encoder can learn the same conceptual abstraction independently from original, non-normalized inputs. Low-consistency detection is evaluated on a held-out human-annotated subset rather than solely on threshold-based similarity labels. This reduces the risk of circular evaluation, although the annotation of semantic consistency may still involve expert judgment. The concept lexicon constitutes another potential source of corpus dependence. Although it was manually curated, fixed before model training, and contained no human consistency labels or model-derived information, its lexical variants were collected from the same five translations evaluated in this study. Consequently, some expressions occurring in the held-out splits were already represented in the normalization resource. This setting does not involve label leakage because no human consistency labels, model predictions, or evaluation results were used. Nevertheless, the normalization resource has access to lexical forms occurring in the held-out data and therefore represents a closed-corpus evaluation setting rather than a strict unseen-corpus setting. The present findings should therefore be interpreted as evaluating a manually curated closed-corpus expert lexicon.

**External validity.** As a Confucian classic, the *Analects* is characterized by concise expression, dense conceptual content, and substantial interpretive openness. These properties make concept-aware semantic consistency modeling particularly suitable for the present task. However, it remains unclear whether the same advantages can be directly generalized to other types of classical texts, modern texts, or corpora with lower concept density. Moreover, the current study focuses only on the comparison of multiple English translations of a Chinese classical text. Its applicability to other language pairs, texts from other cultural traditions, and datasets with different translation formats has not yet been fully validated. The five translations were published between 1861 and 1910 and may not represent modern translation strategies, particularly the retention of romanized concepts such as *ren*, *li*, and *junzi*. Accordingly, under the current evidence, the framework should be understood as a computational aid evaluated within this specific cross-translation setting, rather than as a validated general-purpose method for translation-quality assessment. Future work will compare the present closed-corpus lexicon with externally defined and training-only lexicons to evaluate lexical generalization under stricter unseen-corpus settings.

**Construct validity.** This study operationalizes cross-translation comparison through two primary evaluation tasks, namely retrieval and low-consistency detection. R@1, R@5, and MRR measure retrieval performance, while Accuracy, F1, and AUC measure binary detection performance. Spearman’s ρ and Kendall’s $\tau_{b}$ are reported only as supplementary measures of association between continuous model scores and binary human labels. Because the human annotations contain two categories rather than graded consistency levels, these coefficients should not be interpreted as agreement with a fine-grained semantic ranking. More detailed human annotations would be required to evaluate whether the model captures multiple degrees of semantic consistency.

## Conclusion

This paper proposes a concept-aware semantic consistency modeling framework for multi-translation comparison of the *Analects*. By combining sentence-level alignment, normalization modules, supervised contrastive learning, and concept-aware supervision, the framework learns representations that support cross-translation retrieval and the identification of potentially low-consistency pairs. Experiments show that the proposed method achieves the strongest overall performance across the evaluated tasks. The findings support the framework as a quantifiable and reproducible computational aid for comparing semantic consistency patterns and prioritizing translation pairs for closer expert examination. The framework is not intended to determine overall translation quality or replace interpretive analysis by translation scholars. The moderate association between the model scores and the binary human labels further indicates that the framework should be used to support expert interpretation rather than replace it. These findings are limited to the five historically English translations of the *Analects* examined here and require broader validation. Future work will introduce graded human annotations to evaluate finer levels of semantic consistency.
